# The Relevance and Added Value of Geriatric Medicine (GM): Introducing GM to Non-Geriatricians

**DOI:** 10.3390/jcm10143018

**Published:** 2021-07-07

**Authors:** Marina Kotsani, Evrydiki Kravvariti, Christina Avgerinou, Symeon Panagiotakis, Katerina Bograkou Tzanetakou, Eleftheria Antoniadou, Georgios Karamanof, Athanasios Karampeazis, Anastasia Koutsouri, Kyriaki Panagiotopoulou, George Soulis, Konstantinos Stolakis, Ioannis Georgiopoulos, Athanase Benetos

**Affiliations:** 1 Working Group on the Development of Geriatric Medicine in Greece of the Hellenic Society for the Study and Research of Aging, 15342 Athens, Greece; ev.kravvariti@gmail.com (E.K.); c.avgerinou@ucl.ac.uk (C.A.); simeongpan@hotmail.com (S.P.); k.tzanetakou@external.euc.ac.cy (K.B.T.); antoniadou@upatras.gr (E.A.); gkaramanof@hotmail.com (G.K.); karampeazis@yahoo.gr (A.K.); nkalamak@yahoo.gr (A.K.); kiriakipanagiotopoulou@gmail.com (K.P.); geosoulis@yahoo.com (G.S.); kstol@hotmail.it (K.S.); i.georgiopoulos@chru-nancy.fr (I.G.); a.benetos@chru-nancy.fr (A.B.); 2 Department of Geriatrics, CHRU de Nancy, 54500 Vandœuvre-lès-Nancy, France; 3 FHU CARTAGE-PROFILES, Université de Lorraine, 54505 Vandœuvre-lès-Nancy, France; 4 1st Department of Propaedeutic Internal Medicine, Laiko General Hospital, 11527 Athens, Greece; 5 Postgraduate Medical Studies in the Physiology of Aging and Geriatric Syndromes, School of Medicine, National and Kapodistrian University of Athens, 11527 Athens, Greece; 6 Department of Primary Care and Population Health, University College London, London NW3 2PF, UK; 7 Department of Internal Medicine, Heraklion University Hospital, 71003 Heraklion, Greece; 8 Medical Psychology Unit, School of Medicine, European University Cyprus, 1516 Nicosia, Cyprus; 9 Rehabilitation Unit, Patras University Hospital, 26504 Rio, Greece; 10 Geriatric Clinic, Vrinnevi Hospital, 60379 Norrköping, Sweden; 11 Medical Oncology Unit, NIMTS Veterans Hospital, 11521 Athens, Greece; 12 Henry Dunant Hospital Center, Outpatient Geriatric Assessment Unit, 11526 Athens, Greece; 13 Geriatric Ward, Hôpital “Sainte Thérèse”, VIVALIA-IFAC, 6600 Bastogne, Belgium; 14 School of Social Sciences, Hellenic Open University, 26335 Patras, Greece; 15 School of Medicine, Patras University, 26504 Rio, Greece; 16 INSERM DCAC, 54505 Vandœuvre-lès-Nancy, France

**Keywords:** geriatric medicine, non-geriatricians, clinical practice, added value, geriatric approach

## Abstract

Geriatric Medicine (GM) holds a crucial role in promoting health and managing the complex medical, cognitive, social, and psychological issues of older people. However, basic principles of GM, essential for optimizing the care of older people, are commonly unknown or undermined, especially in countries where GM is still under development. This narrative review aims at providing insights into the role of GM to non-geriatrician readers and summarizing the main aspects of the added value of a geriatric approach across the spectrum of healthcare. Health practitioners of all specialties are frequently encountered with clinical conditions, common in older patients (such as cancer, hypertension, delirium, major neurocognitive and mental health disorders, malnutrition, and peri-operative complications), which could be more appropriately managed under the light of the approach of GM. The role of allied health professionals with specialized knowledge and skills in dealing with older people’s issues is essential, and a multidisciplinary team is required for the delivery of optimal care in response to the needs and aspirations of older people. Thus, countries should assure the educational background of all health care providers and the specialized health and social care services required to meet the demands of a rapidly aging society.

## 1. Introduction

Older people comprise the greatest share of patients seen by general practitioners (GPs) and specialists, excluding pediatricians and obstetricians, and this share is expected to rise. As stated by the WHO in the priorities set for the Decade of Healthy Aging 2020–2030 [1], health systems need to be aligned to the needs of older people and human resources required for the provision of integrated health care must be ensured.

Geriatric Medicine (GM), the medical specialty concerned with the health and wellbeing of older adults, displays a highly variable stage of development across countries, with several Balkan countries lacking a formal recognition of Geriatric Medicine as a medical specialty [2,3]. Consequently, basic principles of GM, essential for dealing with older people, are commonly unknown. Recent research has demonstrated the gap in GM education as the main barrier to providing person-centered care for older people with frailty in the community [4]; nevertheless, it has been observed that physicians and nurses are motivated and receptive to training in geriatric skills with a direct clinical application [5]. Moreover, even in countries with developed GM systems, there is often a vague understanding amongst GPs and specialists of what GM is and what geriatricians do [3,6,7].

The aim of this article is to provide insights into the role of GM to non-geriatrician readers and to summarize the main aspects of the value of a geriatric approach across the spectrum of healthcare.

### Definition, Aim, Means and Tools of GM

As implied by its definition (Table 1), GM ‘s relevance extends throughout the range of health care (primary care, acute care, rehabilitation, long term care) and deals with all aspects of individual health (physical, functional, cognitive, psychological, nutritional, social, etc.). Long before organ-specific guidelines highlighted the need for a person-centered approach [8,9], GM had already focused on multidimensional assessment and individualized management of the problems of older people [10]. Common conditions frequently affecting the geriatric patient (Table 1) go beyond strict disease definitions and conventional management of single pathologies [11]. The goal of GM is to integrate the management of these conditions under the aim of preserving function, maintaining autonomy as long as possible, promoting wellbeing and social engagement, and, at the end of one’s trajectory, humane end-of-life care. Life prolongation is not an absolute goal in GM and aspects such as quality of life; personal values; individualized health priorities; and rational, often modest, objectives frequently guide clinical decisions.

Geriatricians are trained to recognize the variable expressions of aging processes—from the usual (normal) to the accelerated (pathological)—and their impact in functional outcomes. Additionally, geriatricians typically are familiar with the complexity of older patients’ multimorbidity, polypharmacy, atypical disease presentation, uncertainty of indicated management (due to scarcity of clinical guidelines applicable to frail older adults), and increased risk of side effects of treatments and interventions.

In GM, the clinical judgment, aiming at a tailored approach, is combined with the application of standardized procedures. The Comprehensive Geriatric Assessment (CGA) (Table 1) is the standardized tool typically used by the geriatric team to approach complex problems of the geriatric patient. CGA is shared and enhanced by the cooperative work of allied health professionals, such as nurses, psychologists, social workers, physiotherapists, occupational and speech therapists, clinical pharmacists etc. Family and professional caregivers may also be involved in the process. Other medical specialists, especially general practitioners (GPs), play an important role by participating in the CGA, elaborating and applying a coherent action plan, but also in the first step of screening and referral of those eligible to benefit from CGA. A Cochrane review of trials has shown that older patients are more likely to be alive and in their own homes at follow-up if they received CGA on admission to hospital [25]. Through a CGA, the health and wellbeing needs of the older person are identified and a tailored plan of care is elaborated, addressing issues that are of concern to the older person and/or their caregiver. The choice of therapeutic goals should be collaborative and based on the priorities of older people, which are not necessarily identical to those of health professionals [26,27]. Appropriate interventions are then scheduled and progress is reviewed periodically [28].

Ultimately, the GM approach is not exclusively reserved to geriatricians and members of the geriatric team. It is relevant for all health care professionals who deal with older patients’ problems in their daily practice.

## 2. Geriatric Medicine and Community-Based Health Care

Older people wish to remain in their own homes for as long as possible [29], however, complex care needs of community-dwelling older persons make them more prone to rapid deterioration when at home, especially when community-based support is inadequate [30]. The present infrastructure and healthcare paradigms in most countries are not sufficient to meet those needs [29].

Currently, most health care systems respond to geriatric conditions (falls, frailty, cognitive decline, loss of autonomy) at a secondary care level. The importance of continuity of care is undeniable, but at the same time it is also true that health-care systems are designed to deal with single organ disease and clinical guidelines use disease-specific targets [31]. Geriatric patients with complex needs fail to fit in this model, especially those with frailty, thereby warranting a different way to organize primary care.

A move towards a community-based, proactive, integrated, personalized response system is required [31], enabling the management and coordination of complex health needs of older people at a community level and avoiding hospitalizations, falls, cognitive decline, and loss of autonomy [32]. GM’s focus on functional status, personalized needs of the older person, and tailored interventions re-arranges primary care and preventive medicine priorities, assisting the work of GPs, reducing the burden of health care systems and providing real benefit to the older patient. Primary prevention of an adverse cardiovascular event within a 6–7-year timeframe may be less relevant for the 85-year-old frail person at risk of considerable drug-related negative effects on his/her functional status. On the contrary, assessment and monitoring of functional status clinical indices, such as gait speed measurement, could lead to interventions preventing further loss of autonomy and/or timely organization of strategies to meet anticipated growing needs.

Several acknowledged approaches may be applied towards reorganizing community care according to older peoples’ needs and GM principles, and to achieve integration and continuity of older persons’ care across primary care, hospital, and community settings [33]. The British Geriatrics Society has created frailty detection tools and best practice guidelines that may be easily used by primary health care physicians [31,34]. In New Zealand, efforts are being made to organize and assess integrated care models targeted to improve access to specialist assessment and comprehensive care for older people at risk of functional decline with primary care in its center [30] and in creating a primary–secondary care interface for people with complex needs living in the community [35]. Ireland proposes a model of local implementation of Integrated Care Program for Older People where practical guidance is provided to multidisciplinary care teams in the community for older people with complex needs [36]. In France, the Personalized Health Plan for older patients at risk of loss of autonomy (PPS-PAERPA) is a document drawn up jointly under the responsibility of the general practitioner, shared with the patient, and formalizing the action plan for the implementation of multi-professional interventions [37].

Community-based multifactorial interventions linked to a personalized management plan have shown to improve outcomes for older people including reduced nursing home and hospital admissions, reduced falls, and better physical function [32]. CGA in primary care settings has been shown to be acceptable to older people, but with variable outcomes and not enough evidence on which tools are cost-effective, due to the low number of trials in primary care [38].

The overall goal of all these approaches remains the same: keeping older persons safely in place, in harmony with their needs and according to their wishes and values. The personalized, integrated, and functionality-focused approach of GM favors this goal [39], counteracting the mainstream disease-focused approach.

## 3. Relevance of Geriatric Medicine for Non-Geriatrician Specialists

Medical specialists are faced with the need to reconsider their traditional models of care and align them with special needs of the growing population of older patients [11]. Geriatric expertise may aid non-geriatrician specialists manage problems of older patients with a high level of complexity. CGA identifies salient issues and prioritizes the objectives of the therapeutic plan. For example, according to the level of a person’s frailty, some standard treatment options may not be appropriate after consideration of risks and benefits such as chemotherapy for cancer patients with frailty; on the contrary, an 86-year-old patient should not be precluded from consideration for coronary revascularization interventions, merely because of their chronological age [40]. CGA may also contribute to a well targeted pre-habilitation program (Table 1) prior to non-urgent surgery, enhancing post-operative outcomes.

Models of cooperation between geriatricians and other specialists (also known as “co-management” or “co-care” models) [41] have emerged in order to address the complex needs of older patients in acute care, with typical examples such as orthogeriatrics, oncogeriatrics, and intervention of geriatricians in emergency units. However, such models require sufficient numbers of geriatricians and administrative restructuring at the hospital level. An alternative approach could be raising the awareness of non-geriatrician specialists regarding principles of GM. This could facilitate the dialogue between organ specialists and geriatricians and would avoid concrete management of older patients either similarly to younger ones or merely according to their chronological age. Some examples of encounters of organ specialists with geriatric syndromes and principles of GM are discussed below.

### 3.1. Cancer

Cancer is predominantly a disease of the older people with more than 60% of new cases occurring in patients over the age of 65 years [42]. However, older people are underrepresented in the large, randomized trials [43] and, therefore, the applicability of the conclusions of these trials to older cancer patients should be taken with caution.

GM uses prognostication models to identify older adults who are most likely to benefit from screening for a cancer by comparing the calculated life expectancy of the individual with the time to benefit for the cancer treatment and by taking into account the risks of screening tests and overdiagnosis and individual values and preferences [44].

Performance status, typically used to assess the functional status of cancer patients, cannot adequately describe the complexity and the multi-morbidity of the older population [45]. Therefore, CGA, is recommended in order to better define the actual “biological” age and help individualize treatment of older cancer patients [46,47]. The role of such assessment in oncology includes the identification of unrecognized underlying problems that could compromise the patient’s quality of life, complicate cancer treatment, and affect prognosis [46]. Such problems can often be ameliorated with timely interventions if they are identified before cancer treatment starts. In addition, growing evidence suggests that geriatric assessment can predict chemotherapy toxicity and therefore may help make tailored treatment decisions [48,49] and avoid either excess toxicity due to overtreatment or undertreatment. Even though CGA requires geriatric expertise, several screening tools exist for non-geriatrician specialists to identify individuals who could benefit from more in-depth geriatric assessment to optimize cancer management in older patients [50].

### 3.2. Arterial Hypertension

Current convention on hypertension management infers that an intensive treatment of hypertension is always beneficial regardless of the patient’s health, age, frailty, and living conditions. However, the few trials [51,52] conducted in older adults completely excluded patients with clinically significant cognitive decline and dementia, multiple cardiovascular and other co-morbidities, orthostatic hypotension, metabolic disorders, as well as patients with loss of autonomy. For those patients with marked functional decline and loss of autonomy, we only have data from observational studies and most of them show that blood pressure levels less than 130/80 mmHg under antihypertensive treatment are associated with increased morbidity and mortality [53,54,55]. Although presently there are no specific guidelines for the strategies and goals of antihypertensive treatment in the older old patients, it becomes clear that the “one size fits all” concept cannot be applied due to the enormous functional heterogeneity in these individuals [56].

GM proposes an adaptation of therapeutic strategies according to the functional/frailty/autonomy status of these patients [57]: For older patients with preserved functional status, strategies should be those proposed for younger old adults, whereas for those with marked loss of function and autonomy, therapeutic strategies should be thoroughly reassessed, including deprescribing if BP levels are very low [56,57]. Finally, we believe it is necessary to obtain more evidence by including in clinical trials not only over-selected robust older subjects, but also more “typical” geriatric patients (Table 1) [58].

### 3.3. Delirium

Delirium, or acute confusional state, is a common medical problem in older people, which is associated with increased mortality in hospitalized patients (up to 40% in the first year) and poorer outcomes [59]. Approximately 10–16% of patients in emergency departments present with delirium and up to 56% of all hospitalized patients will develop delirium during their stay [60,61], while a fivefold increased risk of delirium is observed when dementia co-exists. Most frequently encountered conditions that can lead to delirium are infections, medication (such as psychoactive and anticholinergic drugs, some analgesics, antibiotics and antiarrhythmics, steroids etc.), drug introduction or withdrawal, metabolic disorders, and organ dysfunction [62,63,64,65]. Delirium should be considered a medical and not a psychiatric problem, which means that the health professionals all across the medical spectrum should be able to correctly identify it, investigate the underlying medical causes of this syndrome, and propose the most adequate therapeutic approach. Nonetheless, non-geriatrician specialists often misdiagnose delirium as dementia or other neurological and psychiatric conditions. Under-recognition of delirium is more possible in its hypoactive form, where glaring symptoms are absent. Misdiagnosis and misconceptions regarding delirium often lead medical teams unfamiliar with this syndrome to the use of inappropriate medication.

Sedative agents may trigger a disastrous cascade of drug adverse events and consequent complications in the older patient with delirium. Non-pharmacological interventions, such as time and place orientation of the patient, early mobilization, medication reconciliation, sleep-wake cycle preservation, sensory impairment restoration, and dehydration treatment are first line measures for the management and, most importantly, for the prevention of delirium [62,63,64,65]. While there are no medications approved by the U.S. Food and Drug Administration (FDA) for the treatment of delirium, antipsychotics such as haloperidol or quetiapine are commonly used to manage symptoms that threaten safety when nonpharmacologic approaches are insufficient. Dexmedetomidine seems to reduce incidence and duration of intensive care unit delirium, while further studies are required to confirm the potential benefit of melatonin in delirium prevention and management [66,67].

Since delirium is often an index of cognitive frailty and its clinical course may be complicated with functional status degradation, a geriatric assessment and tailored intervention is essential for the person’s rehabilitation and discharge plan, and should be sought systematically, in parallel and independently of the management of the initial condition.

### 3.4. Neuro-Cognitive Disorders

Advancing age is associated with increased prevalence of multiple chronic health problems, and at the same time, is characterized by increased odds of cognitive impairment or dementia (also referred as major neurocognitive disorder). The coexistence of multimorbidity and cognitive impairment has a significant impact on the management of chronic diseases, and its timely identification by the therapeutic team is of utmost importance [68]. Most often people with major cognitive disorder also suffer from arterial hypertension (41%), depression (32%), coronary disease (21%), cerebrovascular accident (18%), chronic pain (17%) and diabetes mellitus (13%) [69]. Therefore, encounters with physicians of multiple specialties are not infrequent and several issues related to the impairment of cognitive functions need to be considered. 

Firstly, cognitive deficits affect a person’s ability to receive disease-specific education and practice effective self-management. Timely identification of cognitive impairment can help simplify the therapeutic regimen (e.g., once-daily versus multiple dosing regimens), and/or recommend a health aid or use of assistive equipment (e.g., pill boxes), in order to avoid (or reduce) medication errors. Furthermore, cognitive impairment may affect a person’s ability to describe their symptoms and provide a concise medical history, resulting in a delayed or erroneous diagnosis, and may hinder a person’s ability to follow complex cost/benefit discussions during the informed consent process. Finally, establishing a dementia diagnosis may guide decisions regarding advanced care planning.

Additionally, comorbidity complicates the medical management of dementia: Caution is required when initiating and up-titrating first line therapy with acetylcholinesterase inhibitors in older adults with heart conduction problems, or other organ systems (i.e., prostate enlargement), which are common among the geriatric patient group. Moreover, antipsychotic drugs used to control behavioral problems in older persons with dementia, have been linked to cerebrovascular accidents and cardiac events, especially in older persons with cardiometabolic comorbidities. The CGA provides the vantage point from which the physician can individualize chronic disease management, mobilize social benefits, and shape the patient’s philosophy of care, according to their personal values and prognosis.

### 3.5. Other Neurological Conditions

Geriatricians are often found at the side of neurologists in the field of battle against neurological conditions. Along with standard disease management, the role of the geriatrician in stroke care is to prevent the anticoagulation undertreatment for stroke prevention, to balance the benefits and risks of polypharmacy for cerebrovascular events (primary and secondary) prevention [70]; to advocate against ageistic prejudice regarding stroke treatment decisions; and to anticipate and help manage stroke-related complications such as mental, mobility, and swallowing problems leading to falls and malnutrition, delirium, pressure ulcers, etc. [71]. Geriatricians are there also to recognize symptoms related to microcerebrovascular disease such as urinary incontinence and gait problems, to diagnose epilepsy, or a chronic subdural hematoma during a complete falls’ work up, to assess and limit the risk of osteoporotic fractures in the faller, and to consider the burden of caregivers, particularly challenging in the case of neurological diseases [72]. Moreover, by systematically assessing the gait and balance in their patients, geriatricians often contribute to early diagnosis of neurological diseases, such as Parkinson’s disease and related disorders, normal pressure hydrocephalus and small vessels cerebrovascular disease [73]. A geriatricians’ preoccupation regarding a patient with parkinsonian syndromes expands to their mental and cognitive health, sleep quality, orthostatic hypotension and falls, constipation and urinary retention risk, swallowing problems, and malnutrition.

### 3.6. Mental Health Disorders

Mental health (MH) symptoms often go undiagnosed regardless of the age group and are considerably prevalent in older adults [74,75,76]. There are many challenges in the diagnosis and management of pre-existing MH disorders in the aging adult and in the differential diagnosis from the onset of a neurodegenerative disorder: symptoms of the ageing process, MH problems, and age-related neurodegenerative disorders often overlap [77]. Initial symptoms of major neurocognitive disorders may present as manifestations of other MH disorders such as depression and anxiety [78,79,80,81,82]. At the same time, both depression and anxiety can interfere with cognitive performance [17,83,84,85,86].

MH symptoms often accompany common geriatric conditions and major life stress factors [87,88] such as loss of spouse [89,90], social isolation [91], loneliness [92,93,94], chronic disease [95], unintentional weight loss and malnutrition [96,97,98], the post-fall syndrome [99], physical disability [100], and loss of autonomy [101,102]. Given the dynamic relationship of physical health and MH, the importance of addressing MH symptoms as part of the ageing population’s general health is central.

The GM approach typically includes cognitive and mood assessment, as well as integrated in-depth MH assessments when appropriate, and can identify a suitable management plan to address MH disorders in older adults. Caution should be advised with using medication to treat MH disorders, since, despite their wide use, they can often be inappropriate for older patients, especially the frail ones [103,104].

### 3.7. Rheumatologic and Musculoskeletal Diseases

Rheumatologic and musculoskeletal diseases (RMDs) are common ailments among older persons, and in many cases are major contributors to functional limitation and frailty. Osteoporosis, the hallmark of the aging skeleton, is a leading cause of age-related disability worldwide, and geriatricians are avid advocates of primary and secondary osteoporotic fracture prevention and vigorous post-fracture rehabilitation [105]. The geriatrician’s viewpoint is also significant in the management of osteoarthritis, a typical wear-and-tear process of aging bone and cartilage, especially for patients requiring joint replacement operations in order to maintain mobility and an acceptable quality of life in the final decades of life; for the oldest-old and more complex patients, a geriatric expert can assist with weighing the operative risks against daily suffering, in the context of patient preferences and values. Gout, which may present atypically with oligo- or polyarthritis and involvement of unusual sites in older patients, becomes more common, owing to rising prevalence of polypharmacy and key comorbidities, such as chronic renal insufficiency, or lymphoproliferative and myelodysplastic syndromes, which also limit treatment alternatives [106]. Pseudogout, a typical disease of old age, albeit rarer, should not be overlooked as a cause of large joint monoarthritis or oligoarthritis. The shoulder is commonly affected by calcific tendinitis or rotator cuff injuries, requiring long courses of physical therapy. Suspicion of typical inflammatory RMDs of older adults, such as polymyalgia rheumatica/giant cell arteritis spectrum and late-onset rheumatoid arthritis warrants rheumatologic consultation regardless of age, to avoid the well-recognized risk of undertreatment in this population [107]. Although still insufficient, available data show that novel therapies can be effective and well tolerated in older age groups; individually tailored decisions should incorporate information on patients’ biological age and frailty status, as provided by the CGA [108].

### 3.8. Malnutrition

Protein-energy malnutrition is caused by an imbalance between intake and the body’s requirements. Its prevalence increases with age and is estimated as 4–10% in older persons living at home, 15–38% in those in institutional care, and 30–70% in hospitalized older patients [109]. Of note, isolated protein deficiencies may be observed even in older persons apparently in good health [110,111].

Malnutrition (e.g., undernutrition) in acute care, is associated with disease or injury and is a combination of reduced food and nutrient intake and acute inflammation. These parameters lead to altered body composition and diminished biological function in hospitalized older people.

Involuntary weight loss in older patients leads most physicians to initiate investigations for malignant or chronic inflammatory diseases. Although the above mentioned diseases are common in older adults, several other conditions can also lead to decreased food intake and loss of weight: loss of taste and smell, poor oral health, difficulties in chewing and swallowing, side effects of pharmacological treatment, vision impairment, cognitive and mobility limitations, social isolation, loneliness and depression, and even financial restrictions. Further, in hospitalized or institutionalized patients, external factors such as the “light diet” approach and the quality and appearance of meals may affect dietary intake and contribute to malnutrition. Strict compliance to disease-specific diets is often inappropriate for frail older patients and may lead to disproportional secondary effects; unless associated with a cautious dietary guidance and adequate physical activity, restrictive diets can lead to protein-energy malnutrition causing tissue loss, mostly muscle wasting (i.e., sarcopenia, Table 1), falls, fractures, and devastating consequences for the functionality of older people. Malnutrition is also associated with increased morbidity and mortality both in acute and chronic disease; has severe implications for recovery from disease, trauma and surgery [112]; and leads to more infectious complications, longer hospital stays, an increased rate of readmissions to hospital, and higher health resource utilization [113,114].

### 3.9. Surgical Conditions

The range of problems and particularities of each geriatric, surgical patient needs a broad interdisciplinary and holistic approach and treatment, encompassing the perioperative period and postoperative rehabilitation [115]. Regarding the involvement of geriatricians in the management of surgical patients, most of the current experience is obtained through the cooperation of geriatricians and surgeons according to the orthogeriatric model. The principles of GM and the results of the CGA are also applied in the cases of major oncological and cardiovascular operations.

In the case of a scheduled surgical intervention, the CGA will recognize the individualized care needs of the patient and create a specified plan for them [116]. Moreover, the CGA will identify the persons at increased risk for adverse surgical outcomes, complications, and increased mortality, such as patients with frailty [117,118]. By evaluating the benefit–risk balance from a planned surgical intervention, GCA may sometimes conclude foregoing surgical treatment may be of greater benefit to an older patient, by taking into account their frailty profile, functional status, expected benefits, anticipated risks, and personal value system. In case the expected benefit from the operation is judged more important than the risks, an interdisciplinary team—including a surgeon, an anesthetist, and a geriatrician—organizes a pre-habilitation plan [119], aiming to optimize the patient’s physical and mental capacity prior to surgery. At the same time, the geriatric assessment will aim to identify and manage the various diseases and comorbidities, polypharmacy, and to mitigate factors that may lead to adverse outcomes [115].

GM principles applicable during the peri- and post-operative period are relevant even for emergency surgical interventions in older patients. The geriatric evaluation, by identifying the “profile” of the patient, may provide crucial information leading to a better evaluation of the surgical and anesthetic risk and to the choice of the most appropriate surgical and anesthetic methods [115].

Postoperatively, the role of geriatric care remains important: in the management of pain, delirium, medication, medical problems, and complications; in the assessment of the patient’s mental and nutritional status; in the prevention of pressure ulcers; in ensuring immediate mobilization and rehabilitation; as well as the organization of a discharge plan and postoperative follow-up [115].

Geriatrics is the discipline that ensures the continuation of care for the older patient before, during, and after surgery, helping to optimize outcomes by preventing perioperative complications and reducing hospitalization length and associated costs [120], with the purpose of maintaining or improving functional status [121,122].

## 4. Geriatric Rehabilitation

Geriatric Rehabilitation (GR) (Table 1) aims to restore function or enhance residual functional capability in older people with disabling impairments, particularly those with frailty [123,124]. Current rehabilitation practice focuses on function and well-being, not exclusively on disease [125,126], which is also in alignment with the GM’s core focus.

Several factors in the rehabilitation of older persons distinguish it from the rehabilitation of younger adults, such as the higher burden of comorbidity and the multicausal nature of disability [126]. Their rehabilitation needs, as well as the optimum clinical management, should be identified through the CGA, which may uncover conditions that were previously unrecognized and untreated (level of evidence 1) [126].

Notwithstanding the progressive course of chronic diseases, any acute illness in the geriatric patient can seriously threaten functioning and their autonomy level. Thus, a rehabilitation care plan, of variable intensity and complexity, aiming at functional recovery, turns out to be necessary in almost any geriatric patient case encountered in clinical practice. There is a high level of evidence for the interdisciplinary team approach (level 1), and the management by a physician and team trained in care of older adults (level 1) [127].

At an international level, rehabilitation care for older adults is provided in different settings, such as in hospitals (rehabilitation ward, long-term care hospitals, community hospitals), skilled nursing facilities, care homes, or in ambulatory and home-based settings [128,129,130]. Multidisciplinary geriatric rehabilitation and/or exercise interventions in acute settings decreased mortality, improved physical and functional performance of patients and autonomy in Activities of Daily Living at discharge, and reduced the length of hospital stay and likelihood of being discharged to a nursing home, compared to patients receiving only usual care [131,132,133,134,135].

## 5. Relevance of the Geriatric Approach in the Nursing Care of Older Patients

The geriatric approach of a nurse towards older patients requires more than traditional nursing duties [136]. Geriatric nursing requires specific skills in frail patient care, interdisciplinary treatment planning, rehabilitation, management of neurocognitive problems and geriatric syndromes, tackling psychosocial problems (depression, loneliness, isolation), abuse (physical, psychological and financial), neglect and existential issues, fostering effective communication, and therapeutic education both for patients and their families/caregivers [137].

Geriatric nurses are often those who first get in contact with patient’s needs, identify under-recognized issues, and have a key role in the multidisciplinary team as they are crucial players, along with geriatricians, in coordinating the team, in acting as a liaison between different health care providers, and in taking over a significant part of the follow up, especially in home care settings.

Geriatric nurse duties can be challenging and demanding, and the need for staff trained in dealing with the specific needs of older patients is evident, in view of the scarcity of human resources in the field and the undoubted growth of the older population segment [138].

A clinical case example of the GM approach is presented in Table 2.

## 6. Conclusions and Implications for Practice

Geriatric medicine has a crucial role in promoting health and managing the complex medical, cognitive, social, and psychological issues of older people.

There is remarkable variation between the resources, infrastructure, and geriatric education among different countries. The role of geriatricians is unique and cannot be completely fulfilled by GPs and other specialists, as it requires specific training and clinical experience relevant to this specific population. The role of allied health professionals with specialized knowledge and skills in dealing with older people’s issues is essential and a multidisciplinary team is required for the delivery of optimal care in response to the needs and aspirations of older people.

Nevertheless, all professionals who are concerned with the older peoples’ health should have insights into principles of GM. In countries where Geriatric Medicine is still emerging, the government, health authorities, education institutes, and the scientific community should acknowledge this gap and work towards fulfilling the educational background and specialized health and social care services required to meet the demands of a rapidly aging society.

## Figures and Tables

**Table 1:** Glossary.

Geriatric Medicine	The medical specialty concerned with physical, mental, functional, and social conditions in acute, chronic, rehabilitative, preventive, and end of life care in older patients [12].
Geriatric patient	The geriatric patient is an older patient typically presenting the following characteristics: reduced homeostatic ability, atypical presentation of diseases, multimorbidity and polymedication, psychosocial vulnerability, and geriatric syndromes, including frailty and (risk of) loss of autonomy [13]. Typically, the conventional chronological age corresponding to the “older patient” is 65 years. However, beneficiaries of geriatric services are most often people aged 75 years and older.
Geriatric team	A multidisciplinary team of health professionals who run the comprehensive geriatric assessment and implement the therapeutic plan. Members of the geriatric team usually, but not exclusively, are geriatricians, nurses, social workers, dietitians, physiotherapists, occupational therapists, speech therapists, and clinical pharmacists. Other professionals, such as dentists and other medical specialists, can also be involved on demand.
Comprehensive Geriatric Assessment (CGA)	A multidimensional, interdisciplinary diagnostic process to determine the medical, psychological, and functional capabilities of an older person with frailty, followed by implementation of a coordinated and integrated plan for treatment and follow-up [6]. CGA is typically a stepwise approach that uses standardized tools and methods to initially assess several (ideally all) dimensions of the health of an older person (both frailties and assets) and is subsequently completed by in-depth investigations and targeted management of detected problems.
Geriatric Syndromes	Multifactorial health conditions, common to older adults, that occur when the accumulated effects of impairments in multiple systems render an (older) person vulnerable to situational challenges [14]. A geriatric syndrome primarily refers to one symptom or a complex of symptoms with high prevalence in geriatrics, resulting from multiple diseases, multiple risk factors, and interacting pathophysiological mechanisms [15]. Examples include falls, cognitive disorders and delirium, frailty, sarcopenia, malnutrition, pressure ulcers, sphincter incontinence, polypharmacy etc.
Frailty	A clinically recognizable state in which the ability of older people to cope with every day or acute stressors is compromised by an increased vulnerability brought by age-associated declines in physiological reserve and function across multiple organ systems [16]. A person with frailty typically responds less favorably even to a mild stressor (e.g., a trivial infection, a psychological stress, an environmental change etc.) compared to a person without frailty, with more pronounced decrease in its functional capacity (at a system and organism level), a slower recovery, and a diminished capacity for full recovery. Although the most recognizable type of frailty is physical frailty (characterized by diminished strength, endurance, and reduced physiologic function), there are also other types of frailty described such as cognitive frailty, social frailty etc.
Delirium	Delirium or acute confusional state according to DSM V * comprises a common medical problem in older people, characterized by the acute onset of deficits in attention, awareness, and cognition that fluctuate in severity over time. Additional features include psychomotor disturbance, altered sleep cycle, and emotional variability. According to the psychomotor disturbances, delirium is distinguished into three phenotypic subtypes. (a) Hyperactive delirium manifested with psychomotor agitation, restlessness, and emotional instability is sometimes misdiagnosed as primary psychosis or mania. (b) Hypoactive delirium exhibits psychomotor retardation, lethargy, and decreased level of responsiveness, and is often misdiagnosed as depression. (c) Mixed delirium presents with alternating features of the above types [17].
Sarcopenia	Sarcopenia is a progressive and generalized skeletal muscle disorder that is associated with increased likelihood of adverse outcomes including falls, fractures, physical disability, and mortality. Sarcopenia is probable when low muscle strength is detected. A sarcopenia diagnosis is confirmed by the presence of low muscle quantity or quality. When low muscle strength, low muscle quantity/quality and low physical performance are all detected, sarcopenia is considered severe [18].
Orthogeriatrics	Orthogeriatrics is the subspeciality area in geriatrics involved in the care of older people with fragility, mostly hip fractures. It also involves pre-operative assessment and management of patients undergoing elective hip, knee, and spinal surgery. Orthogeriatrics was developed as a subspecialty to address the poor outcomes of hip fracture patients by caring for patients alongside orthopedic surgeons and with the support of a specialist multidisciplinary team [19,20].
Oncogeriatrics	Oncogeriatrics or geriatric oncology is a branch of medicine that is concerned with the diagnosis and treatment of cancer in the older patients. The oncogeriatric approach tends to classify patients in terms of their frailty, aiming at proposing appropriate oncological treatments and to associate geriatric interventions for the prevention and management of geriatric syndromes and related risks [13].
Pre-habilitation	The multimodal approach aiming at the optimal preparation of a patient for a major intervention, by increasing physical, physiological, metabolic, and psychosocial reserves and enhancing general health and well-being. It incorporates numerous systemic and regional approaches that include exercise, nutrition, education, and/or psychosocial interventions that are intended to improve preoperative fitness and preparedness. Importantly, this also promotes and facilitates health behavior changes not only preoperatively but during the postoperative period and long-term [21,22,23].
Geriatric Rehabilitation	A multidisciplinary set of evaluative, diagnostic, and therapeutic interventions with the purpose to restore functioning or enhance residual functional capability in older people with disabling impairments [24].

* DSM V: Diagnostic and Statistical Manual of Mental Disorders (fifth edition), [17].

**Table 2:** Clinical case scenario presenting a rather common case in everyday practice according to the standard management without a “geriatric medicine insight” vs the geriatric medicine approach.

The Scenario	Standard Vision and Critical Management Points	The Geriatric Medicine’s Point of View
A male 82-year-old patient, living with his wife and autonomous regarding the basic daily activities of daily living, is admitted to a non-geriatric hospital ward with an altered level of consciousness and breathlessness. His wife, going back home from the usual visit to the grocery shop, called for help after finding him lying on the ground, conscious but unable to get up. His glasses are broken. He usually walks with a walking stick. His wife reports a moderate loss of appetite during the past few days, a generalized fatigue and weakness, slightly incoherent sayings and a breathlessness in effort. In the last 3 months he has had another two falls but managed to get up immediately.	There is probably an acute medical problem that explains this alteration in the physical state of this gentleman. It is not rare for an old person to stumble and fall for random reasons.	The patient already presents an initial status of possible frailty, at least at a physical level and maybe some signs of reduced autonomy: he uses a walking aid, is autonomous for the basic activities of daily living but it is his wife that does the shopping, he falls repeatedly. Frailty on the cognitive level may also be suspected by the acute cognitive decompensation due to the acute illness. Frailty renders him more prone to short- and long-term adverse outcomes in case of acute illness. The recent fall is a serious event, especially if unable to get up on his own. Questions rise about his nutritional and hydration state these last days.
His medical history includes arterial hypertension, dyslipidemia, chronic obstructive pulmonary disease (COPD), stage IIIa chronic kidney disease, chronic lumbar and knee aches, and an anxiety-depressive syndrome. His usual weight is 78 kilos for a height of 1.67 m (Body Mass Index, BMI 28kg/m^2^). His usual medical treatment includes a combination of inhaled corticosteroids and long-acting-beta-agonist, simvastatin, perindopril, amlodipine, alopurinol, aspirin, pantoprazole, escitalopram, paracetamol in case of pain, and oral corticoids in case of acute exacerbation of his COPD. His COVID-19, influenza, and pneumococcal vaccination are up to date.	The patient represents a rather common case of an older patient treated for multiple comorbidities. Since his BMI is >25kg/m^2^, he is considered being overweight. He is treated with medications for all of his comorbidities.	There is no clear indication for the use of alopurinol, aspirin, and pantoprazole. Escitalopram may raise the risk of falls, similarly to antihypertensive drugs in case of hypotension. Could pain have contributed to this risk of fall? Could he be suffering from sarcopenia (due to reduced physical activity, inadequate protein intake, corticosteroid treatment, statin muscle waste…)? Regardless of his BMI, what is his nutritional status?
On admission, the patient presents: confusion with time and space disorientation, diffuse wheezing and crackles at right base, symmetrically swollen ankles, moderate desaturation on air, and a temperature of 38.1 degrees. Blood pressure 105/60 mmHg, pulses 95/min. No serious traumatic injury following his fall, nor focal neurological deficit are observed. The laboratory analysis is characterized by an inflammatory syndrome. Tests for viral pulmonary infections are negative. His electrocardiogram is normal and his brain imaging without significant abnormality for the age.	No acute heart pathology or stroke are found. No traumatic consequence from the fall. Fall-related work up is checked completed. Diagnosis: probable pulmonary infection, acute COPD exacerbation, and possibly heart decompensation.	An acute condition, fever with respiratory tract infection, has decompensated a previous frail status and requires treatment. A rehabilitation plan needs to be elaborated from the beginning and in parallel with his acute condition treatment. A full fall work up needs to be performed as soon as possible. Multiple falls always need further investigation and corrective actions.
On the third day of hospitalization under antibiotic and corticosteroid treatment, oxygen therapy, bronchodilators, and diuretics, the patient develops an acute confusional state with agitation, aggressiveness, and opposition to treatment, requiring restraint. Clinical exam reveals no abnormal findings other than some crackles on the right pulmonary basis and signs of urinary retention. Blood testing reveals an improvement of the inflammatory syndrome, normal oxygen and CO2 levels, moderate hyperglycemia, and an elevation of creatinine and sodium levels. A bladder catheter is placed and a treatment with haloperidol IM and alprazolam is initiated. Physical restrain is prescribed on demand in case of significant agitation and opposition to treatments.	Older people frequently present acute psychiatric conditions while hospitalized. Could it be dementia? Physical restraint and pharmacological seduction are judged necessary for the patient’s safety and compliance to treatments.	Delirium is often mistaken for dementia, even though they frequently overlap. Urinary retention, corticosteroid therapy, electrolyte disorders, anticholinergic agents such as bronchodilators, and environmental factors such as transfer in an unfamiliar environment and sensory deficits (visual impairment) may trigger a delirium. Physical restraint often exacerbates agitation, especially when inappropriately administered. The risk of urinary retention would be lower if early mobilization had been achieved. First line treatment of delirium is non-pharmacological approaches and elimination of trigger factors. Currently, there is no good evidence showing whether or not benzodiazepines should be used for the treatment of delirium [139]. Even though the relatively short stay of the patient on the ground during his fall did not lead to significant muscle damage, oxygen therapy, bladder catheter, physical restraint, and a lack of systematic mobilization of the patient result in an excessive bedrest and in a deconditioning state of psychomotor and functional decline.
Progressive rehydration and improvement of the confusional state occurs after almost 5 days. During this time, neuroleptics are continued, and the patient presents a daytime somnolence, due to which he skips meals and visits of the physiotherapist. Physical restraint is applied in the daytime to limit the risk of fall. He feels very weak and with a low appetite. He now weighs 73.5 kilos.	Regarding physical restraint in the daytime, “it’s better being safe than sorry”, since the patient presents now an obvious risk of falling again. Weight lost is considered “beneficial” for the patient’s cardio-metabolic profile and his joint aches.	Nutritional support and mobilization need to be scheduled early and promptly implemented during the process of the acute disease management, along with the evaluation of swallowing problems, risk assessment, and prevention of pressure sores and of delirium. Loss of 4.5 kilos corresponds to more than 5% of the patient’s weight lost in less than a month, which already diagnoses a state of malnutrition in a person > 70 years old. Sedative medication should be discontinued as soon as possible.
The patient’s medical chart and blood tests improve progressively and, after 13 days of hospitalization, he is discharged in a wheelchair with a low salt and sugar diet, tamsulosin, furosemide, and lorazepam added to his prior medication list and advice for the care of a heel pressure sore.	Low salt is recommended as a non-pharmacological measure against hypertension, whereas a low sugar diet is recommended because corticoid-induced hyperglycemia and overweight are considered prodromal signs of diabetes. Three new drugs are added and maintained at discharge: Furosemide due to ankle oedema, tamsulosine under the hypothesis of a prostatic hypertrophy that contributed to the urinary retention and lorazepam because of sleeping problems and circadian rhythm inversion.	Extreme caution should be applied before recommending restrictive diets in frail older people. Risk of malnutrition, sarcopenia, and loss of autonomy usually outweigh anticipated benefits from dietary adaptations that aim at managing cardiovascular risk factors. Restrictive diets bear the risk of inadequate protein intake, which, on the other hand, is essential for recovery and pressure sore healing. It is suggested that older persons with acute and chronic diseases should have an intake of 1.2–1.5 g of protein per kilogram body weight [140]. Long term furosemide treatment is rarely required and the possibility of amlodipine contributing to ankle oedema should be considered. The indication of a tamsulosin treatment is not well documented and may cause hypotensive episodes, especially in association with multiple antihypertensive medication. Sleep hygiene interventions should be used as first line treatment of sleeping disorders. Benzodiazepines increase the risk of falling. Our patient now has several fall risk factors: multiple medication, frailty and probably sarcopenia, prior walking difficulties and fall history, visual deficit, and an aching heel pressure sore. A comprehensive rehabilitation and aid plan should be conducted before going back home, including physiotherapy for gait, balance and muscle strength, nutritional support, and adaptation of the supportive environment.
One month after discharge, the patient is operated under general anesthesia for a hip fracture after a new fall. According to his wife, the last month the patient presented more cognitive difficulties than usual, required aid in toileting and feeding, with occasional swallowing problems, dizziness, gait difficulties, and repeated falls. The patient is discharged from the orthopedics department to a nursing home in a worse state of confusion, bedridden with a bladder catheter and a with major weight loss (66 Kg).	A fall resulting in a hip fracture is an event that can lead to the institutionalization of an older patient	A succession of unfavorable outcomes in a patient presenting an underlying frailty resulted in a downward spiral that ended up in a loss of autonomy. Many of these outcomes were preventable or could have been managed in an earlier and more appropriate way, according to the approach and principles of Geriatric Medicine. Loss of autonomy could have been delayed or prevented.

## Data Availability

No new data were created or analyzed in this study. Data sharing is not applicable to this article.
